# Effects of *Prunella vulgaris* polysaccharide-based O/W emulsions on water retention, protein conformation, and antioxidant properties of ultrasonically treated reduced-fat pork batter

**DOI:** 10.1016/j.ultsonch.2026.108078

**Published:** 2026-09-23

**Authors:** Jing-Chao Fan, Kai Wang, Guang-Hui Liu, Wen-Bin Ma, Zhuang-Li Kang

**Affiliations:** a School of Pharmacy, Shangqiu Medical College, Shangqiu 476100, PR China; b Key Laboratory of Chinese Cuisine Intangible Cultural Heritage Technology Inheritance, Ministry of Culture and Tourism, School of Tourism and Cuisine, Yangzhou University, Yangzhou 225127, PR China

**Keywords:** *Prunella vulgaris*, Secondary structure, Texture, Raman, TBARS

## Abstract

This study examined how *Prunella vulgaris* polysaccharides (PVP, 0, 2% and 4% w/w in the O/W emulsion) and ultrasonic treatment (0 and 30 min) affect key quality attributes of reduced-fat pork batter. Combined PVP and ultrasound significantly improved (*P* < 0.05) emulsifying stability and water-holding capacity, cutting cooking loss from 11.75% to 5.88%. PVP alone enhanced water–protein–polysaccharide interactions: it shortened T_21_ and T_22_ relaxation times, raised immobilized water from 86.18% to 93.95%, and lowered overall water mobility. It also altered myofibrillar protein structure, reducing α-helix and increasing β-sheet, β-turn, and random coil content, which correlated with significant improvements (*P* < 0.05) in L* value, hardness, springiness, cohesiveness, chewiness, and sensory scores for color, texture, and overall acceptability. PVP suppressed lipid oxidation during 7-day refrigerated storage (lower TBARS, *P* < 0.05), though ultrasound partially weakened this effect. Overall, using PVP-based O/W emulsions as pork back-fat replacers, and applying ultrasound, is a promising strategy to simultaneously improve water retention, gel network formation, and oxidative stability in reduced-fat emulsified pork products.

## 1 Introduction

Emulsified meat products constitute a diverse and economically accessible category widely favored by consumers. Traditionally, these products contain relatively high levels of animal fat, typically exceeding 20%, which critically contributes to their characteristic flavor, juiciness, and desirable texture [1], [2]. However, chronic overconsumption of saturated fats is epidemiologically linked to elevated risks of obesity, cardiovascular disease, and other metabolic disorders [3]. Direct fat reduction compromises structural integrity, leading to inferior textural attributes (e.g., softness, crumbliness), diminished flavor release, and reduced water retention [4], [5]. To circumvent these drawbacks, a well-established approach involves partial substitution of animal fat with plant-based O/W emulsions formulated from plant proteins, unsaturated vegetable oils, and water [6]. Although effective in lowering saturated fat content, such emulsions often suffer from physical instability (e.g., creaming, coalescence) and heightened susceptibility of unsaturated lipids to oxidative degradation, thereby accelerating quality deterioration and shortening shelf life [7]. To address both challenges simultaneously, functional biopolymers, particularly plant-derived polysaccharides endowed with dual emulsion-stabilizing and antioxidant capacities, are increasingly explored as multifunctional food-grade additives [8].

*Prunella vulgaris L*., a perennial herb of the Lamiaceae family, is a renowned traditional Chinese medicinal herb with documented use dating back to “Shennong Ben Cao Jing” (The Divine Farmer’s Materia Medica). It is widely distributed across East Asia and Europe and valued for its rich phytochemical profile and broad-spectrum bioactivities [9]. *Prunella vulgaris* polysaccharides (PVP) are heterogenous, high-molecular-weight glycoconjugates primarily composed of arabinose, rhamnose, galactose, glucose, xylose, and mannose [10]. Their structural complexity, including branched architectures and dynamic higher-order conformations, underpins diverse physiological functions, notably potent antioxidant, anti-inflammatory, and immunomodulatory activities [11]. Mechanistically, PVP exhibit free radical scavenging capacity against superoxide anion (O_2_•^−^), hydroxyl (•OH), and nitric oxide (•NO) radicals; inhibit lipid peroxidation in cellular and model systems; and demonstrate ferric ion (Fe^3+^) chelating ability and reducing power, collectively confirming robust antioxidant potential [12], [13]. In food applications, PVP have been successfully incorporated into beverages, breads, cakes, and biscuits to enhance nutritional density and functional performance. However, the application of PVP in muscle-based foods, especially emulsified meat products, remains limited.

Ultrasound technology, an emerging non-thermal physical processing method, exerts its effects primarily through acoustic cavitation, characterized by the formation, growth, and violent collapse of microbubbles in a liquid medium [14], [15]. This collapse generates localized transient conditions of extreme temperature (> 5000 K) and pressure (> 100 MPa), along with intense microstreaming and microjetting forces [15]. These combined thermal and mechanical stimuli can effectively disrupt non-covalent interactions within myofibrillar proteins, inducing partial unfolding of the native conformation and thereby enhancing protein solubility and surface hydrophobicity [16]. Crucially, cavitation-induced structural perturbation exposes previously buried functional groups, including hydrophobic amino acid residues and free sulfhydryl (–SH) groups, facilitating their rapid migration to the oil–water interface [15]. This interfacial adsorption reduces interfacial tension and promotes the formation of a cohesive, viscoelastic protein film around oil droplets, which is essential for achieving high emulsifying activity and long-term emulsion stability [17]. Prior optimization study identified 40 kHz frequency, 192 W ultrasonic power, and 30 min treatment duration as the most effective parameters for improving gel strength and water-holding capacity in pork batter [18], [19], establishing a robust foundation for the current investigation. Hence, this study develops O/W emulsions with PVP (0%, 2%, or 4% w/w, relative to total emulsion mass) as partial replacements for pork back-fat in reduced-fat pork batters, followed by standardized ultrasonication. We comprehensively evaluate how these PVP-based emulsions affect water retention, gel network integrity, sensory attributes, and oxidative stability of the ultrasonically treated, reduced-fat pork resulting batters.

## 2 Materials and methods

### 2.1 Materials and ingredients

Fresh *longissimus dorsi* muscle (24 h post-mortem; moisture: 71.42%, protein: 19.73%, fat: 6.07%, pH = 5.76 ± 0.01) and pork back-fat (fat: 92.32%, moisture: 6.52%) were procured from a local farmers’ market (Shangqiu, China). Soy protein isolate (protein ≥ 91.36%) was supplied by Shandong Shansong Soy Foods Co., Ltd. (Jining, China). Both meat and fat were ground separately using a laboratory meat grinder fitted with a 6-mm plate, vacuum-packaged in nylon/PE laminated bags, and stored at − 40 °C until use; total frozen storage duration did not exceed 4 weeks. Refined soybean oil (fat content: 99.90%) was purchased from Shanghai Standard Foods Co., Ltd. (Shanghai, China). *Prunella vulgaris* L. was cultivated in the Pharmacy Botanical Garden of Shangqiu Medical College (34°21′18.76″N, 115°42′23.72″E; soil type: yellow loam). Sodium tripolyphosphate, salt, and commercial spice blend were obtained from a local supermarket (Shangqiu, Henan Province, China). All chemical reagents were of analytical grade.

### 2.2 Extraction of PVP and preparation of O/W emulsions

PVP was extracted following a modified version of the protocol described by Zhao et al. [20]. Briefly, dried aerial parts of *Prunella vulgaris* L. were defatted with 95% ethanol (8 × volume, 12 h, room temperature), filtered, and the residue was air-dried. The defatted material was then subjected to hot-water extraction three times (1:10 g·mL^−1^ solid-to-liquid ratio, 100 °C, 2 h each), and the combined extracts were concentrated under reduced pressure (≤ 50 °C). Ethanol was added to the concentrate to achieve a final concentration of 80% (v/v), followed by cold precipitation at 4 °C for 24 h. The precipitate was collected by centrifugation (2000 × g, 10 min, 4 °C), washed with anhydrous ethanol, and lyophilized to yield crude PVP powder.

According to the method of Kang et al. [21], O/W emulsions were formulated with soy protein isolate as the primary emulsifier. The base formulation consisted of soy protein isolate: deionized water:soybean oil = 1:6:6 (w/w/w). PVP was incorporated at 0%, 2%, or 4% (w/w, relative to total emulsion mass), the PVP replacement formulas used the same weight of water and oil (water: oil = 1:1). Emulsion preparation proceeded as follows: soy protein isolate and PVP were dispersed in deionized water and hydrated under magnetic stirring (room temperature, 2 h). Soybean oil was then added gradually, and the mixture was homogenized using an Ultra-Turrax T25 (IKA, Staufen, Germany) at 10,000 rpm for 2 min. The resulting emulsion was stored at 4 °C for 12 h to allow structural equilibration prior to use.

### 2.3 Preparation of reduced-fat pork batter

Three experimental formulations were prepared, each containing: 500 g lean pork meat, 100 g pork back-fat, 100 g ice water, 2 g sodium tripolyphosphate, 8 g salt, 5 g spice blend, and 100 g O/W emulsion. Among them, the pork batters containing 0%, 2%, and 4% PVP O/W emulsions were named as C (0% PVP, w/w), T1, (0.25% PVP, w/w) and T2 (0.50% PVP, w/w) respectively. Frozen pork meat were thawed under refrigeration (4 °C) for 12 h until the center temperature reached ≤ 4 °C. The processing protocol was as follows: Thawed lean pork was placed in a bowl chopper (Stephan UMC-5C, Hamburg, Germany) and mixed with salt and sodium tripolyphosphate at 1500 rpm for 60 s. Subsequently, half of the ice water was added, and mixing continued at 1500 rpm for another 60 s. Finally, the remaining ice water, spice blend, pork back-fat, and O/W emulsion were incorporated sequentially and homogenized at 1500 rpm for 60 s. Throughout the entire mincing process, the chopper bowl was immersed in an ice–water bath to maintain the temperature of the meat batter below 7 °C. Upon completion, 200 g freshly prepared batter (approximately 200 cm^3^) was vacuum-packaged in nylon/PE pouches (batter size was approximately 20 cm × 10 cm × 1 cm) and subjected to ultrasonic treatment under standardized conditions: frequency 40 kHz, power output 192 W, acoustic power density 12.8 W/L, duration 30 min. Ultrasonication was performed using an ultrasonic machine (KM-615D, Guangzhou Komeng Clean Technology Co., Ltd., China) in an ice–water bath (maintained at 0–4 °C) to prevent thermal denaturation; the resulting samples were labeled UC (0% PVP), UT1 (0.25% PVP), and UT2 (0.50% PVP). For gel formation, 40 g of raw batter was weighed into a 50 mL centrifuge tube, degassed by centrifugation (2000 × g, 3 min, 4 °C) to eliminate entrapped air bubbles, then heated in a circulating water bath at 80 °C for 20 min. The cooked gels were cooled to ambient temperature (25 ± 2 °C), followed by refrigerated storage at 4 °C prior to analysis.

### 2.4 Emulsification stability

Emulsification stability was assessed according to Fernandz-Martín et al. [22]. Approximately 20 g of raw batter was placed in a pre-weighed 50 mL centrifuge tube and heated in a water bath at 80 °C for 20 min (center temperature confirmed as 72 ± 2 °C). After cooking, the tube was removed, rinsed under tap water until the sample surface temperature equilibrated to 25 ± 2 °C, and then inverted over a pre-weighed beaker lined with qualitative filter paper to collect exuded fluid. The beaker containing the expressed juice was weighed, dried at 105 °C for 16 h, and re-weighed. Total release, fat release, and water release were calculated as follows:

$$\text{Total release}\;\left(\%\right)=\left[\left({\text{W}}_{2}-{\;\text{W}}_{1}\right)/\;\text{W}\right]\times \;\text{1}00$$

$$\text{Fat release}\;\left(\%\right)=\left[\left({\text{W}}_{2}-{\;\text{W}}_{1}-{\;\text{W}}_{3}\right)/\;\text{W}\right]\times \;\text{1}00$$

$$\text{Water release}\;\left(\%\right)=\left[\left({\text{W}}_{3}-{\;\text{W}}_{1}\right)/\;\text{W}\right]\times \;\text{1}00$$

where W = initial weight of raw batter (g); W_1_ = tare weight of empty beaker (g); W_2_ = weight of beaker + expressed juice (g); W_3_ = weight of beaker + dried residue (g).

### 2.5 Cooking loss determined

According to the section 2.3, the cooked pork batters were prepared. Cooking loss was calculated as:

$$\text{Cooking loss}\;\left(\%\right)=\left(\text{Weight of raw batter}\;-\;\text{Weight of cooked pork batter}\right)/\;\text{Weight of raw batter}\;\times \;\text{1}00$$

### 2.6 Color determined

Cooked pork batters were cut into cylindrical specimens (2 cm height, 2.5 cm diameter). Color parameters (L*, a*, and b* values) were measured at the central cross-section using a calibrated colorimeter (CR-400, Konica Minolta, Japan) equipped with an 8-mm aperture, 10° standard observer angle, and D65 illuminant.

### 2.7 Texture properties determined

Cooked pork batters were tempered at 25 ± 2 °C for 2 h, and then cut into uniform cylinders (2 cm diameter × 2 cm height). Texture properties were determined using a texture analyzer (Stable Micro Systems, UK) fitted with a P/36R cylindrical probe. Test parameters: pre-test speed 5 mm/s; test speed 2 mm/s; post-test speed 2 mm/s; compression strain 50%. The hardness, springiness, cohesiveness, and chewiness of cooked pork batters were obtained.

### 2.8 Low-field nuclear magnetic resonance (LF-NMR)

Samples were prepared following Kang et al. [4]. Cooked pork batters were cut into approximately 2 g cubes, placed in 15 mm outer-diameter glass NMR tubes, and analyzed using a PQ001 LF-NMR spectrometer (Niumag Electric Corporation, China). Measurements were conducted at 32 °C with a proton resonance frequency of 22.6 MHz. Pulse sequence: 90°–τ–180°–τ–echo; τ = 200 μs; number of scans = 32; repetition time = 6.5 s; total echo count = 12,000.

### 2.9 Raman spectroscopy analysis

The Raman spectroscopy analysis of cooked pork batter was conducted following the method of Kang et al. [23]. The parameters were as follows: 600 g.mm^−1^ grating, slit 200 μm, 3 scans of data, acquisition speed of 120 cm^−1^.min^−1^, resolution of 2 cm^−1^, and integration time of 60 s.

### 2.10 Sensory evaluation

Cooked pork batters were cut into 1 cm^3^ cubes and served in white porcelain dishes under controlled lighting (D65 daylight simulator). Ten trained panelists (aged 22–45 years, with ≥ 100 h sensory training in meat products) evaluated each sample for color, texture, juiciness, and overall acceptability using a 9-point hedonic scale (1 = extremely dislike; 9 = extremely like). Sensory training contained subject recruitment and screening, environmental standardization training, scale usage norms, and bias prevention mechanism.

### 2.11 Lipid oxidation

Lipid oxidation in cooked pork batter was quantified using the thiobarbituric acid reactive substances (TBARS, mg MDA/kg) assay, adapted from Zhu et al. [24]. TBARS values were determined on days 0, 1, 4, and 7 of refrigerated storage.

### 2.12 Statistical analysis

All experiments were conducted in triplicate (n = 3) using independently prepared batches of pork batter, with variation introduced by both PVP concentration levels and ultrasonic treatment duration. Data are presented as mean ± standard error (SE). Statistical analyses were performed using the GLM procedure in SPSS v.26.0 (IBM, USA). Fixed effects included PVP addition level and processing method (with or without ultrasonication), while replicate was treated as a random effect. Mean comparisons were conducted using the LSD test; Duncan’s Multiple Range Test was applied post hoc to identify statistically significant differences among treatment means at *P* < 0.05.

## 3 Results and discussion

### 3.1 Emulsifying stability

Emulsification stability reflects the capacity of meat batter to retain fat and water during thermal processing, serving as a key quality indicator for meat products. As shown in Table 1, both total release and water release in pork batters decreased significantly (*P* < 0.05) with increasing PVP incorporation. Fat release exhibited an initial decline followed by stabilization. This behavior is primarily attributed to the strong water absorption and retention capacity of PVP, which forms hydrogen bonds with water molecules, thereby mitigating water evaporation during heating, and simultaneously encapsulates fat globules, suppressing their coalescence and rupture under thermal stress, ultimately enhancing emulsification stability [13]. Moreover, ultrasonic treatment disrupts the polysaccharide structure via cavitation, improving the processability of myofibrillar proteins and promoting synergistic interactions between proteins and PVP; this facilitates the formation of a more robust gel network with enhanced binding affinity for both water and fat [25], [26]. Consequently, all ultrasonically treated formulations exhibited lower total release, water release, and fat release compared with their non-sonicated counterparts. Within the sonicated group, total liquid and water losses declined progressively with increasing PVP concentration, whereas fat loss plateaued after an initial reduction. Notably, incorporating PVP conferred comparable stabilization of fat globules in the O/W emulsion; however, the higher PVP formulation achieved superior water retention in pork batter.

**Table 1:** The effects of preparing O/W emulsion from the *Prunella vulgaris* polysaccharide and ultrasonic treatment on the emulsifying stability (total release, fat release, and water release) of reduced-fat pork batter.

Sample	Total release (%)	Water release (%)	Fat release (%)
C	10.53 ± 0.28^a^	8.25 ± 0.19^a^	2.26 ± 0.16^a^
T1	8.79 ± 0.31^b^	7.52 ± 0.14^b^	1.27 ± 0.17^b^
T2	7.33 ± 0.26^c^	6.35 ± 0.16^c^	1.08 ± 0.13^b^
UC	9.16 ± 0.19^b^	7.81 ± 0.15^b^	1.35 ± 0.14^b^
UT1	6.59 ± 0.27^d^	5.77 ± 0.13^d^	0.82 ± 0.16^c^
UT2	5.63 ± 0.26^e^	4.92 ± 0.15^e^	0.71 ± 0.14^c^

Each value represents the mean ± SE, n = 3.

^a-e^ Different Parameter superscripts indicate significant differences (*P* < 0.05).

### 3.2 Cooking loss

Cooking loss directly influences product juiciness and flavor, and thus constitutes a critical determinant of consumer acceptability. As presented in Fig. 1, cooking loss in pork batter decreased progressively (*P* < 0.05) with higher PVP addition levels. This trend arises from PVP-induced elevation of O/W emulsion viscosity, which enhances system stability and minimizes thermally driven water and fat leakage [12]. Ultrasonic treatment further reduced cooking loss across all PVP concentrations relative to untreated controls, and the magnitude of reduction intensified with increasing PVP dosage, a pattern consistent with that observed for total juice loss (Table 1). Mechanistically, ultrasound-induced cavitation promotes covalent and non-covalent cross-linking between PVP and muscle proteins, facilitating the development of a denser, more cohesive heat-induced gel network with improved water-holding capacity, thereby limiting juice exudation during cooking [25], [27]. These findings align with those of Kang et al. [18], who demonstrated that ultrasonic treatment strengthens protein gel architecture and significantly reduces cooking loss.

**Fig. 1:**
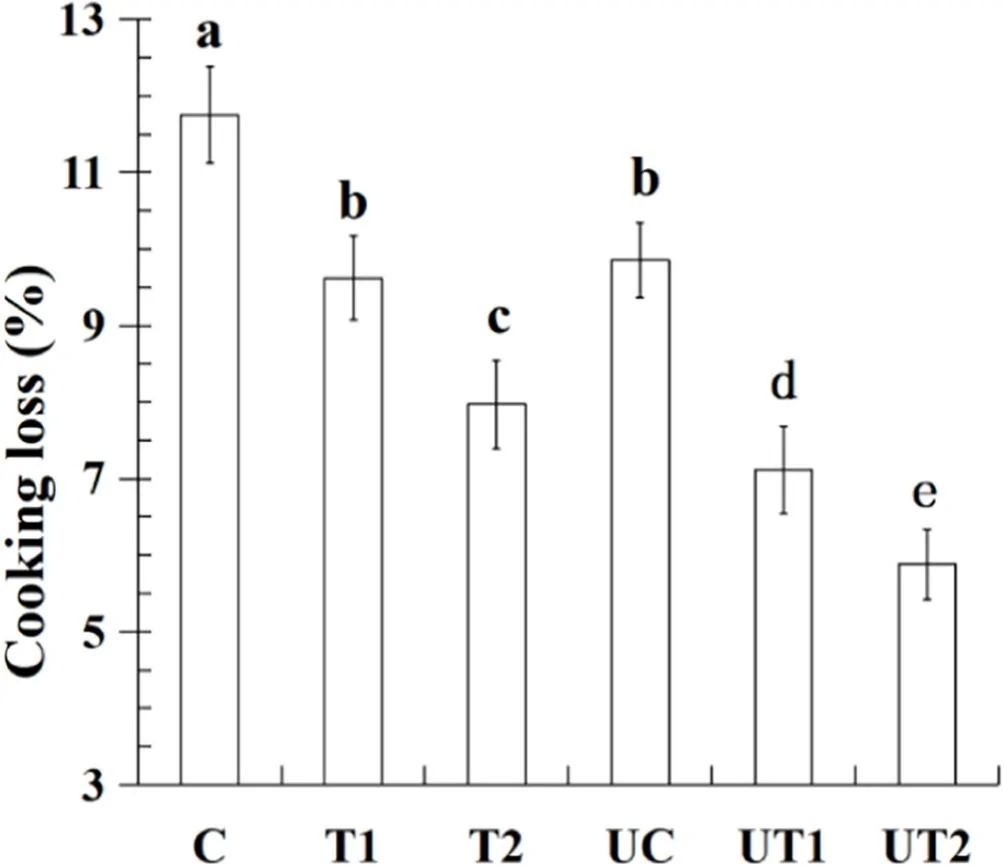
The effects of preparing O/W emulsion from the *Prunella vulgaris* polysaccharide and ultrasonic treatment on the cooking loss of reduced-fat pork batter. Each value represents the mean ± SE, n = 3. ^a–e^ Different Parameter superscripts indicate significant differences (*P* < 0.05).

### 3.3 Color

Color is the foremost sensory attribute guiding consumer evaluation of meat product quality and a decisive factor influencing market acceptance and purchase intent. As presented in Table 2, the L* value of cooked pork batter increased significantly (*P* < 0.05) with increasing PVP concentration, whereas the a* value decreased significantly (*P* < 0.05); the b* value exhibited an initial significant decline (*P* < 0.05), followed by stabilization. These changes stem from PVP’s water-binding capacity: higher moisture increases surface light reflectance and L* value, while diluting myoglobin and lowering both a* and b* values [1], [28]. Ultrasonic treatment further amplified these effects: all sonicated formulations showed significantly higher L* and b* values (*P* < 0.05) and a significantly lower a* value (*P* < 0.05) relative to their non-sonicated counterparts. Within the sonicated group, L* value increased progressively with PVP dosage; a* declined consistently; and b* value followed the same biphasic pattern significant decrease (*P* < 0.05) then plateau. Mechanistically, ultrasound improves the solubility and emulsifying activity of myofibrillar proteins, facilitates intimate polysaccharide, protein integration, suppresses phase separation, and stabilizes the pigment dispersion, collectively enhancing color uniformity, brightness, and overall visual quality [29]. Notably, prior studies [4], [23] have demonstrated that synergistic ultrasonic treatment and polysaccharide incorporation effectively elevate L* value in meat systems, corroborating the observed brightness enhancement.

**Table 2:** The effects of preparing O/W emulsion from the *Prunella vulgaris* polysaccharide and ultrasonic treatment on the color (L*、a* and b* values) of cooked reduced-fat pork batter.

Sample	L* value	a* value	b* value
C	73.52 ± 0.30^d^	4.57 ± 0.15^a^	9.29 ± 0.31^b^
T1	75.16 ± 0.34^c^	3.81 ± 0.14^bc^	8.35 ± 0.29^c^
T2	76.75 ± 0.33^b^	3.54 ± 0.19^c^	7.27 ± 0.32^d^
UC	74.83 ± 0.35^c^	4.02 ± 0.13^b^	10.46 ± 0.34^a^
UT1	77.04 ± 0.31^b^	3.16 ± 0.15^d^	9.11 ± 0.33^b^
UT2	78.59 ± 0.29^a^	2.83 ± 0.12^d^	8.13 ± 0.32^c^

Each value represents the mean ± SE, n = 3.

^a-d^ Different Parameter superscripts indicate significant differences (*P* < 0.05).

### 3.4 Texture properties

Texture properties critically determine consumer perception of palatability and directly govern product acceptability. As shown in Table 3, incorporation of PVP alone significantly improved (*P* < 0.05) hardness, springiness, cohesiveness, and chewiness of cooked pork batter, attributable to PVP’s intrinsic water-holding capacity and gel-strengthening functionality. In contrast, no significant differences of T1 and T2 (*P* > 0.05) were observed across all four texture parameters, indicating insufficient PVP–protein interaction without physical activation [12], [26]. Among ultrasonically treated samples, hardness and chewiness increased significantly (*P* < 0.05) with rising PVP concentration; springiness and cohesiveness, while elevated relative to controls, did not differ significantly across PVP levels (*P* > 0.05). This suggests that ultrasound enables effective PVP dispersion and promotes robust complexation with soy protein isolate, reinforcing the gel matrix and mitigating phase separation, thereby markedly enhancing structural integrity [27]. Consistently, ultrasonicated formulations exhibited significantly superior hardness, springiness, cohesiveness, and chewiness versus non-sonicated controls (*P* < 0.05). Underlying this improvement is ultrasound-induced enhancement of salt-soluble protein solubility and promotion of protein cross-linking, both of which consolidate the heat-induced gel network and augment mechanical resistance to deformation [17]. These findings align with established evidence that ultrasonic treatment strengthens the myofibrillar protein gel network, thereby improving key textural attributes, including hardness and springiness, in meat paste systems [19].

**Table 3:** The effects of preparing O/W emulsion from the *Prunella vulgaris* polysaccharide and ultrasonic treatment on the texture properties of cooked reduced-fat pork batter.

Sample	Hardness (N)	Springiness	Cohesiveness	Chewiness (N.mm)
C	52.63 ± 0.84^e^	0.842 ± 0.007^c^	0.556 ± 0.019^c^	24.66 ± 0.21^e^
T1	55.42 ± 0.76^c^	0.895 ± 0.006^ab^	0.613 ± 0.024^b^	30.40 ± 0.27^cd^
T2	56.89 ± 0.93^c^	0.887 ± 0.010^b^	0.622 ± 0.014^b^	31.39 ± 0.28^c^
UC	54.81 ± 0.79^cd^	0.873 ± 0.008^b^	0.619 ± 0.017^b^	29.62 ± 0.30^d^
UT1	58.26 ± 0.92^b^	0.915 ± 0.009^a^	0.680 ± 0.021^a^	36.24 ± 0.25^b^
UT2	61.33 ± 0.86^a^	0.896 ± 0.008^ab^	0.693 ± 0.018^a^	38.08 ± 0.23^a^

Each value represents the mean ± SE, n = 3.

^a-e^ Different Parameter superscripts indicate significant differences (*P* < 0.05).

### 3.5 LF-NMR

LF-NMR provides insight into water distribution and mobility within meat matrices. In cooked pork batter, transverse relaxation times T_2b_, T_21_, and T_22_ correspond respectively to bound water, immobilized water, and free water. Bound water refers to water tightly associated with macromolecules (e.g., proteins and polysaccharides) via hydrogen bonding or hydration shells; immobilized water resides within the micropores of the protein–polysaccharide gel network and exhibits restricted mobility; free water is bulk-like, unbound, and freely diffusible [4], [30]. As shown in Table 4, initial T_2b_ values showed no significant differences across treatments (*P* > 0.05), indicating consistent binding of water to macromolecular surfaces. In contrast, both T_21_ and T_22_ decreased significantly with increasing PVP concentration (*P* < 0.05), suggesting enhanced restriction of water mobility, particularly for immobilized and free water fractions, due to stronger interactions with the gel matrix. Shorter T_2_ relaxation times reflect tighter physical confinement and reduced molecular mobility, corroborating improved water retention capacity [31]. This trend aligns with concurrent improvements in emulsion stability (Table 1) and reductions in cooking loss (Fig. 1). The underlying mechanism likely involves hydrophilic functional groups (e.g., hydroxyl and carboxyl) in PVP forming hydrogen bonds with water molecules, thereby augmenting water adsorption and entrapment within the gel network [13]. Ultrasonic treatment further shortened T_21_ and T_22_ values relative to non-sonicated controls (*P* < 0.05), indicating that ultrasound intensifies water–matrix interactions, possibly by promoting structural reorganization and densification of the gel network, thereby enhancing its capacity to immobilize water [15].

**Table 4:** The effects of preparing O/W emulsion from the *Prunella vulgaris* polysaccharide and ultrasonic treatment on the initial relaxation time (ms) and peak ratio (%) of cooked reduced-fat pork batter.

Sample	Initial relaxation time (ms)			Peak ratio (%)		
	T_2b_	T_21_	T_22_	P_2b_	P_21_	P_22_
C	1.02 ± 0.09^a^	58.56 ± 1.93^a^	790.05 ± 27.61^a^	1.13 ± 0.10^a^	86.18 ± 0.46^d^	12.63 ± 0.18^a^
T1	0.95 ± 0.11^a^	53.87 ± 1.84^b^	713.58 ± 28.80^b^	0.95 ± 0.11^a^	89.73 ± 0.51^c^	8.36 ± 0.22^b^
T2	0.98 ± 0.10^a^	49.42 ± 2.16^c^	635.60 ± 30.19^c^	0.97 ± 0.09^a^	92.60 ± 0.53^b^	6.41 ± 0.20^c^
UC	0.99 ± 0.13^a^	54.60 ± 2.25^b^	692.37 ± 26.37^b^	1.02 ± 0.08^a^	90.06 ± 0.47^c^	8.84 ± 0.19^b^
UT1	1.06 ± 0.12^a^	49.31 ± 2.04^c^	603.52 ± 29.52^cd^	0.90 ± 0.10^a^	92.47 ± 0.38^b^	6.59 ± 0.17^c^
UT2	1.07 ± 0.10^a^	44.83 ± 2.18^d^	533.61 ± 28.44^e^	0.94 ± 0.07^a^	93.95 ± 0.49^a^	4.06 ± 0.21^d^

Each value represents the mean ± SE, n = 3.

^a-e^ Different Parameter superscripts indicate significant differences (*P* < 0.05).

The corresponding peak areas of P_2b_, P_21_, and P_22_ represent the relative proportions of bound, immobilized, and free water, respectively. While P_2b_ remained unchanged across treatments (*P* > 0.05), P_21_ increased significantly (*P* < 0.05) and P_22_ decreased significantly (*P* < 0.05) with rising PVP dosage, confirming a shift of free water toward the immobilized state, a transformation facilitated by the gel network’s enhanced water-fixing capability. Similarly, ultrasonic treatment significantly increased P_21_ and decreased P_22_ compared with non-sonicated counterparts (*P* < 0.05), reinforcing ultrasound’s role in promoting water redistribution from free to immobilized states. Collectively, these findings demonstrate that the incorporation of PVP, ultrasonic treatment, or the combined effect of both can promote the conversion of free water into immobilized water within cooked pork batter, thereby improving overall water-holding capacity and product quality.

### 3.6 Secondary structure

As shown in Table 5, incorporation of PVP significantly altered the secondary structure composition of myofibrillar proteins in cooked pork batter. Specifically, the α-helix content decreased markedly (*P* < 0.05), whereas β-sheet, random coil, and β-turn contents all increased significantly (*P* < 0.05), likely due to hydrogen bonding between hydroxyl/carboxyl groups of PVP and polar residues on protein surfaces, which disrupts native helical packing and promoting structural reorganization. Notably, PVP concentration exerted no further significant effect on α-helix, random coil, or β-turn contents beyond the lowest tested level (*P* > 0.05), suggesting a saturation threshold for polysaccharide–protein interaction; structural perturbations plateau once optimal interfacial coverage or cross-linking density is achieved [23]. In contrast, β-turn content exhibited a dose-dependent increase (*P* < 0.05), consistent with its role as a nucleation site for gel network formation: enhanced β-turn formation facilitates tighter protein–protein and protein–polysaccharide associations, thereby improving gel order, thermal stability, and mechanical strength. Indeed, β-turn content correlated positively with instrumental cooked pork batter hardness (Table 3), supporting its functional significance in texture development [32], [33]. Ultrasonic treatment amplified these structural shifts: compared with non-sonicated controls, sonicated samples showed significantly higher β-turn and random coil contents and significantly lower α-helix content (*P* < 0.05). This reflects ultrasound-induced partial protein unfolding, driven by cavitation-mediated disruption of intramolecular hydrogen bonds and hydrophobic interactions, which exposes buried hydrophobic patches and reactive side chains, thereby facilitating conformational transitions toward β-turn and disordered states [34]. Such increased structural flexibility enhances compatibility and co-assembling capacity between myofibrillar proteins and PVP, promoting denser, more homogeneous gel networks.

**Table 5:** The effects of preparing O/W emulsion from the *Prunella vulgaris* polysaccharide and ultrasonic treatment on the secondary structure (α-helix, β-sheet, β-turn, random coil, %) of cooked reduced-fat pork batter.

Sample	α-helix	β-sheet	β-turn	Random coil
C	52.74 ± 2.37^a^	18.67 ± 0.36^d^	17.29 ± 0.44^c^	11.34 ± 0.18^c^
T1	47.62 ± 1.92^b^	20.25 ± 0.41^c^	19.50 ± 0.39^a^	12.61 ± 0.22^b^
T2	45.30 ± 2.12^bc^	23.13 ± 0.39^b^	19.06 ± 0.42^a^	12.55 ± 0.21^b^
UC	48.89 ± 2.27^b^	19.74 ± 0.34^c^	18.42 ± 0.45^b^	12.96 ± 0.23^b^
UT1	43.57 ± 2.53^c^	22.32 ± 0.42^b^	19.49 ± 0.47^a^	14.53 ± 0.20^a^
UT2	42.25 ± 1.86^c^	24.73 ± 0.37^a^	18.85 ± 0.41^a^	14.82 ± 0.22^a^

Each value represents the mean ± SE, n = 3.

^a-d^ Different Parameter superscripts indicate significant differences (*P* < 0.05).

### 3.7 Sensory evaluation

As presented in Table 6, PVP addition had no significant effect on color scores of non-sonicated cooked pork batters (*P* > 0.05), whereas ultrasonic treatment significantly improved color scores across all PVP-containing formulations (*P* < 0.05). This aligns with instrumental color measurements (Table 1), which showed increased L* value after sonication, indicating that ultrasound enhanced surface uniformity and brightness, rendering samples visually more appealing to panelists. The lack of color response to PVP alone suggests that polysaccharide-induced optical changes were subtle and below perceptual thresholds without physical modification of the matrix. Texture scores increased significantly upon PVP incorporation (*P* < 0.05), yet remained insensitive to PVP dosage (*P* > 0.05), implying that even minimal PVP levels sufficed to modulate rheological behavior via hydration and network reinforcement. Critically, ultrasonic treatment further elevated texture scores significantly relative to non-sonicated counterparts within each formulation (*P* < 0.05), corroborating improvements in instrumental hardness, springiness, and chewiness (Table 3) and confirming synergistic enhancement of mouthfeel and structural integrity in reduced-fat systems. Juiciness scores showed no significant differences among treatments (*P* > 0.05), likely because PVP forms thermally stable hydrogels during cooking that effectively immobilize moisture, minimizing expressible fluid loss and yielding uniformly perceived succulence across samples [12]. Overall acceptability scores rose significantly with PVP addition (*P* < 0.05), and while scores trended higher in sonicated groups, the difference did not reach statistical significance (*P* > 0.05), indicating that both PVP and ultrasound independently contribute to hedonic quality, but their combined effect, though numerically superior, may require larger sensory panels or refined scaling methods for detection. Collectively, the sample of UT1 and UT2 delivered the highest mean overall acceptability score and optimal balance of instrumental and sensory attributes, supporting its selection as the preferred formulation for reduced-fat pork batter.

**Table 6:** The effects of preparing O/W emulsion from the *Prunella vulgaris* polysaccharide and ultrasonic treatment on the sensory evaluation scores of cooked reduced-fat pork batter.

Sample	Color	Texture	Juiciness	Overall acceptability
C	6.65 ± 0.28^b^	6.82 ± 0.18^c^	6.70 ± 0.26^ab^	6.98 ± 0.22^c^
T1	6.92 ± 0.25^ab^	7.54 ± 0.22^b^	7.14 ± 0.31^a^	8.16 ± 0.25^b^
T2	7.09 ± 0.22^ab^	7.40 ± 0.20^b^	6.93 ± 0.24^a^	7.87 ± 0.23^b^
UC	6.87 ± 0.21^b^	7.03 ± 0.15^bc^	6.91 ± 0.28^a^	7.22 ± 0.31^c^
UT1	7.43 ± 0.23^a^	8.26 ± 0.21^a^	7.27 ± 0.30^a^	8.47 ± 0.26^a^
UT2	7.62 ± 0.27^a^	8.11 ± 0.19^a^	7.16 ± 0.27^a^	8.35 ± 0.28^a^

Each value represents the mean ± SE, n = 3.

^a-c^ Different Parameter superscripts indicate significant differences (*P* < 0.05).

### 3.8 TBARS

The TBARS assay serves as a widely accepted indicator of lipid oxidation extent in meat systems. As shown in Table 7, incorporation of PVP significantly reduced TBARS values in cooked pork batter in a dose-dependent manner (*P* < 0.05), with the greatest inhibition observed in the sample of T2 and UT2. This demonstrates PVP’s potent antioxidant activity, attributable to its capacity to scavenge free radicals (e.g., hydroxyl and peroxyl radicals), chelate pro-oxidant metal ions (e.g., Fe2^+^/Cu2^+^), and inhibit propagation of lipid peroxidation chain reactions, thereby delaying oxidative rancidity and preserving product shelf life [13], [24]. These findings are consistent with prior reports confirming the robust radical-scavenging and lipid-peroxidation-inhibitory activities of PVP-derived phenolic conjugates and reducing sugars [35]. Contrary to expectations, however, ultrasonic treatment resulted in significantly higher TBARS values in PVP-fortified samples versus non-sonicated controls (*P* < 0.05). This apparent pro-oxidative effect likely stems from transient cavitation-induced generation of localized reactive oxygen species (ROS) and mild protein fragmentation during sonication, exposing previously shielded lipid moieties and increasing susceptibility to oxidation during subsequent storage. Nevertheless, all sonicated samples still exhibited substantially lower TBARS values than the control (no PVP, no ultrasound), confirming that PVP’s antioxidant efficacy remains dominant even under ultrasonic stress. Thus, while ultrasound enhances functional properties, its application should be optimized to fully harness synergistic benefits without compromising oxidative stability [36].

**Table 7:** The effects of preparing O/W emulsion from the *Prunella vulgaris* polysaccharide and ultrasonic treatment on the TBARS values (mg MDA/kg) of cooked reduced-fat pork batter during cold storage.

Sample	0 d	1 d	4 d	7 d
C	0.96 ± 0.02^Cb^	1.03 ± 0.04^Cb^	1.35 ± 0.03^Bb^	2.14 ± 0.02^Ab^
T1	0.87 ± 0.03^Cc^	0.92 ± 0.02^Cc^	1.17 ± 0.03^Bc^	1.66 ± 0.03^Ac^
T2	0.79 ± 0.02^Cd^	0.81 ± 0.04^Cd^	0.94 ± 0.05^Be^	1.37 ± 0.04^Ad^
UC	1.13 ± 0.04^Ca^	1.11 ± 0.03^Ca^	1.48 ± 0.04^Ba^	2.39 ± 0.04^Aa^
UT1	0.97 ± 0.05^Cb^	1.02 ± 0.02^Cb^	1.24 ± 0.05^Bc^	1.72 ± 0.02^Ac^
UT2	0.86 ± 0.03^Cc^	0.88 ± 0.03^Cc^	1.06 ± 0.02^Bd^	1.41 ± 0.03^Ad^

Each value represents the mean ± SE, n = 3.

^A–C, a–d^ Different Parameter superscripts indicate significant differences (*P* < 0.05).

As can be seen from Table 7, no significant differences in TBARS were observed among treatments at day 0 or day 1 (*P* > 0.05), reflecting minimal oxidative initiation during initial refrigeration. A significant rise (*P* < 0.05) emerged by day 4, peaking at day 7, indicating progressive accumulation of oxidation products over time. This temporal pattern reflects both autoxidative chain reactions and, in later stages, microbially mediated oxidation: as spoilage microorganisms proliferate during extended refrigeration, their metabolic activity (e.g., enzyme secretion, redox cycling) further accelerates lipid degradation. Collectively, these results demonstrate that while ultrasonic treatment induces a short-term oxidative perturbation, PVP delivers robust, concentration-dependent antioxidant protection that effectively mitigates lipid oxidation throughout refrigerated storage [12], [37], particularly under combined application where functional enhancement and oxidative stability are jointly optimized.

## 4 Conclusion

This study demonstrated that incorporating PVP into pork batter, particularly when combined with ultrasonic treatment significantly reduced total release, water release, and cooking loss. Concurrently, the L* value, hardness, springiness, cohesiveness, and chewiness were significantly enhanced. Correspondingly, the initial relaxation times T_21_ and T_22_ significantly decreased, the peak ratio P_21_ significantly increased, and P_22_ significantly decreased, indicating that ultrasonic and PVP synergistically enhanced the binding ability of water in the gel. Additionally, the incorporation of PVP, ultrasonic treatment, or the combined effect of both led to a significant increase in β-sheet, random coil and β-turn content, indicating that the secondary structure of protein changed and the gel network became more stable. The sensory evaluation results showed that the combination of PVP and ultrasonic treatment significantly improved the color, texture and overall acceptance score of cooked pork batter. During refrigeration, PVP effectively inhibited the lipid oxidation degree of cooked pork batter. In conclusion, using PVP to prepare O/W emulsions to replace pork back-fat and combining with ultrasonic treatment can significantly improve the texture and sensory characteristics of reduced-fat pork batter, and delay the lipid oxidation process.

## CRediT authorship contribution statement

**Jing-Chao Fan:** Writing – review & editing, Writing – original draft, Resources, Project administration, Investigation, Funding acquisition, Conceptualization. **Kai Wang:** Validation, Software, Resources, Formal analysis, Conceptualization. **Guang-Hui Liu:** Writing – original draft, Visualization, Project administration, Methodology, Data curation. **Wen-Bin Ma:** Writing – original draft, Supervision, Software, Investigation, Data curation. **Zhuang-Li Kang:** Visualization, Supervision, Software, Investigation, Data curation.

## Declaration of competing interest

The authors declare that they have no known competing financial interests or personal relationships that could have appeared to influence the work reported in this paper.

## References

[1] Xie C., Liu G.H., Liang M.H., Li S.H., Kang Z.L. (2024). Applying resistant starch to improve the gel and water retention of reduced-fat pork batter. Gels.

[2] Zhang J., Li D., Zhang Y., Tang J., Shi S., Zeng X., Chen H., Pang J., Wu C. (2025). The effects of soy protein isolate-based composite gels as pork back fat substitutes in low-fat emulsified sausage. Food Res. Int..

[3] H.E. Bays, C. Varney, L. Alexander, J. Ng, J. Tondt, C. Weber, S. Christensen, K. Burridge, C. Francavilla, S. Bindlish, B. Shetye, M. Censani, Obesity and cardiovascular disease: an obesity medicine association (OMA) clinical practice statement (CPS) 2026. Obesity Pillars (2026), 100283. 10.1016/j.obpill.2026.100283 PMC13564139 42729919

[4] Kang Z.L., Xie J.J., Li Y.P., Song W.J., Ma H.J. (2023). Effects of pre-emulsified safflower oil with magnetic field modified soy 11S globulin on the gel, rheological, and sensory properties of reduced-animal fat pork batter. Meat Sci..

[5] Xu Y., Qi J., Qi L.W., Liu J.M., Zhao X.R., Chisoro P., Yang P., Zhang C.H. (2025). Agroindustrial waste-derived insoluble dietary fibers as solid fat substitutes for meat emulsion gels: feasibility, modification strategies, and dual-filling mechanisms. Food Bioproc. Tech..

[6] Zhu Z.S., Yang Y., Wang H., Zhang X.H., Li G.L., Huang M. (2026). Beyond animal proteins: insights into the functionality, quality impact, and innovation trends of plant-based proteins in emulsified sausages. Food Res. Int..

[7] Zhu X., Tu Y., Zhao Y., Wu N., Yao Y., Chen S., Wei T., Mao J., Hu X., Wang S., Xu L. (2025). Emulsion gel-based fat replacers in meat products: structured design, processing stability, oral lubricity/flavor perception, and digestive characteristics. Food Chem..

[8] Fu J., Zhan Z., Duan Q., Yang Y., Xie H., Dong X., Zhang H., Yu L. (2025). Application of soybean protein isolates-polysaccharides hybrid emulsion gels as alternative fats in fabricating plant-based meats with two-phase. LWT Food Sci. Technol..

[9] Wang K., Wan Z., Ou A., Liang X., Guo X., Zhang Z., Wu L., Xue X. (2019). Monofloral honey from a medical plant, Prunella Vulgaris, protected against dextran sulfate sodium-induced ulcerative colitis via modulating gut microbial populations in rats. Food Function.

[10] Chen Y., Zhang X., Guo Q., Cao L., Qin Q., Li C., Zhao M., Wang W. (2019). Plant morphology, physiological characteristics, accumulation of secondary metabolites and antioxidant activities of *Prunella vulgaris* L. under UV solar exclusion. Biol. Res..

[11] Li C., Fu X., Huang Q. (2015). Ultrasonic extraction and structural identification of polysaccharides from Prunella vulgaris and its antioxidant and antiproliferative activities. Eur. Food Res. Technol..

[12] Ai X., Yu P., Hou Y., Shu B., Han B., Yang M., Fan X., Wang J. (2025). Research progress in the extraction, purification, structural features, biological activities, and structure-activity relationships from *Prunella vulgaris* polysaccharides. Int. J. Biol. Macromol..

[13] Bi H., Teng W., Wang J., Wang X., Zhang Z., Wang M. (2025). Extraction and purification, structural characteristics, pharmacological activities, structure-activity relationships, applications, and quality assessments of Prunella vulgaris L. polysaccharides: a review. Int. J. Biol. Macromol..

[14] Kang Z., Hou Q., Xu J. (2024). Research progress and teaching exploration of physical processing technology for reduced-salt gel meat products. Foods.

[15] Zhang J., Toldrá F., Kang D.C., Zhou L., Wang J.Y., Zhang W.G., Hu Y.H. (2025). Benefits of ultrasonic technology application in meat field and its influential mechanism: a review. Crit. Rev. Food Sci. Nutr..

[16] Li K., Fu L., Zhao Y.Y., Xue S.W., Wang P., Xu X.L., Zhou G. (2020). Use of high-intensity ultrasound to improve emulsifying properties of chicken myofibrillar protein and enhance the rheological properties and stability of the emulsion. Food Hydrocoll..

[17] Barretto T.L., Sanches M.A.R., Pateiro M., Lorenzo J.M., Telis-Romero J., Barretto A.C.D. (2023). Recent advances in the application of ultrasound to meat and meat products: physicochemical and sensory aspects. Food Rev. Intl..

[18] Kang Z., Shang X., Li Y., Ma H. (2022). Effect of ultrasound-assisted sodium bicarbonate treatment on gel characteristics and water migration of reduced-salt pork batters. Ultrason. Sonochem..

[19] Wu Z., Shang X., Hou Q., Xu J., Kang Z., Ma H. (2024). Using ultrasonic-assisted sodium bicarbonate treatment to improve the gel and rheological properties of reduced-salt pork myofibrillar protein. Meat Sci..

[20] Zhao H., Yang Y., Zhou Y., Wen H., Chen C., Li C., Feng Y., Li L., Li X. (2024). The antihyperplasia effect of polysaccharide from *Prunella vulgaris* L. on mammary gland hyperplasia in rats through regulation of the AKT-FOXO3a signaling pathway and intestinal flora. J. Sci. Food Agric..

[21] Kang Z.L., Chen F.S., Ma H.J. (2016). Effect of pre-emulsified soy oil with soy protein isolate in frankfurters: a physical-chemical and raman spectroscopy study. LWT Food Sci. Technol..

[22] Fernandz-Martín F., Lopez-Lopez I., Cofrades S., Colmenero F.J. (2009). Influence of adding sea spaghetti seaweed and replacing the animal fat with olive oil or a konjac gel on pork meat batter gelation. Potential protein/alginate association. Meat Sci..

[23] Kang Z.L., Kong L.H., Hu Z.L., Li Y.P., Ma H.J. (2023). Effect of sodium bicarbonate and sodium chloride on protein conformation and gel properties of pork myofibrillar protein. Arab. J. Chem..

[24] Zhu M.M., Peng Z.Y., Lu S. (2019). Physicochemical properties and protein denaturation of pork longissimus dorsi muscle subjected to six microwave-based thawing methods. Foods.

[25] Bernardo Y.A.d.A., do Rosario D.K.A., Conte-Junior C.A. (2023). Principles, application, and gaps of high-intensity ultrasound and high-pressure processing to improve meat texture. Foods.

[26] Li J., Zhou Y., Li Z., Ma Z., Ma Q., Wang L. (2023). Mechanism for improving the gel properties of transglutaminase-mediated porcine myofibrillar protein by ultrasonic pretreatment combined with carrageenan. Food Chem..

[27] Zhou W., Sun X., Mo J., Wu M., Yu H., Wang Q. (2025). New insights into the role of ultrasonication in the myofibrillar protein-chlorogenic acid interaction under non-oxidative or oxidative environments and thermal gelation. Food Chem..

[28] Zhuang X.B., Jiang X.P., Han M.Y., Kang Z.L., Zhao L., Xu X.L., Zhou G.H. (2016). Influence of sugarcane dietary fiber on water states and microstructure of myofibrillar protein gels. Food Hydrocoll..

[29] Hu N., You L., Han X., Wang S., Qi W., Xiu L., Cai D. (2025). Effects of different polysaccharide contents on the gel properties of transglutaminase-induced ultrasonic-assisted peanut isolate proteins-corn silk polysaccharide products and their influence on riboflavin release behavior. Food Chem.: X.

[30] Santos D.D.E., Bernardo Y.A.D., Conte C.A. (2026). Unravelling the impact of high-intensity ultrasound on the water mobility of meat products by LF-NMR. Sustainable Food Technol..

[31] Fan J., Liu G., Wang K., Xie C., Kang Z. (2023). Effects of potassium bicarbonate on gel, antioxidant and water distribution of reduced-phosphate silver carp surimi batter at cold storage. Gels.

[32] Bai R., Xi J., Han H.Y., Aidana R., Madina A., Jiang S.Q., Ye X., Yang C.J., Wang S.Y., Ding W. (2025). Mechanistic study of ultrasound synergy with soybean 11S globulin to improve myofibrillar protein gel properties in low-salt lamb: molecular conformation and water migration. Food Res. Int..

[33] Zhu C.Z., Tian Y.D., Liu H., Zhao G.M., Ma Y.Y., Bai X.Y., Wang K. (2024). Having their cake and eat it too: effects of different cations in reduced-salt myofibrillar protein microgel pickering emulsion under high-intensity ultrasound. LWT Food Sci. Technol..

[34] Gómez-Salazar J.A., Galván-Navarro A., Lorenzo J.M., Sosa-Morales M.E. (2021). Ultrasound effect on salt reduction in meat products: a review. Curr. Opin. Food Sci..

[35] Xi Y., Wu Y., Chen G. (2010). Study on isolation and anti-oxidant activity of the polysaccharide from Prunella vulgaris L. J. Guangdong Pharm. Univ..

[36] Sohrabpour S., Kenari R.E., Amiri Z.R. (2020). Effect of cinnamon ultrasound-assisted extract on chemical and microbial properties of hamburger meat under different temperatures and time conditions during storage. J. Food Process. Preserv..

[37] Shi X., Zhang X., Yin J., Nie S. (2014). Antioxidant activities of soluble polysaccharides from *Prunella vulgaris Linn*. J. Food Saf. Qual..

